# Editorial: Diabetes and bone - from cell to human

**DOI:** 10.3389/fendo.2022.1085953

**Published:** 2022-11-17

**Authors:** Peter Vestergaard, Antonino Catalano, Jakob Starup-Linde

**Affiliations:** ^1^ Steno Diabetes Center North Denmark, Aalborg University Hospital, Aalborg, Denmark; ^2^ Unit of Geriatrics, Department of Clinical and Experimental Medicine, University of Messina, Messina, Italy; ^3^ Department of Endocrinology and Internal Medicine, Aarhus University Hospital, Aarhus, Denmark; ^4^ Steno Diabetes Center Aarhus, Aarhus University Hospital, Aarhus, Denmark

**Keywords:** bone, diabetes, fractures, alendronate, antidiabetics medication

The interaction between increased glucose levels and fluctuations in these as seen in diabetes is complex. Changes at the cellular level – even within minutes – may have profound effects on the clinical level leading to an increased risk of fractures. The treatments used to counteract hyperglycemia can modify the effects at the cellular, individual and population levels, but they cannot completely undo the negative effects of the hyperglycemic state. Differences in insulin levels and insulin resistance may influence the effects of hyperglycemia between type 1 and type 2 diabetes but may also play a role within type 2 diabetes, where different phenotypes may exist.

This topic spans the levels from observational studies on the cut-off levels for an effect of HbA1c on bone turnover (Joad et al.) and prevalence of morphometric vertebral fractures in diabetes and pre-diabetes (Hulten et al.) over interventional studies on the effect of diet on bone turnover (Fuglsang-Nielsen et al.) to pharmacoepidemiological studies on fracture risk related to various drugs used in diabetes (Al-Mashhadi et al., Al-Mashhadi et al., Zhang et al., Viggers et al.), even antiosteoporotic therapy may modify the risk of developing diabetes, showing the potential bone pancreas interplay (Viggers et al.), thus demonstrating the necessity to analyse the problem of the bone fragility in diabetes using many different techniques from different fields of science. This also shows that although epidemiological techniques can answer research questions that cannot be answered by preclinical and *in vitro* studies, such as the interaction between diabetes, its treatment, and fracture risk, preclinical studies can elucidate the mechanisms underlying the clinical problem of fractures, which cannot be understood in detail using epidemiology.

On the other hand, interventional studies point out that although understanding and describing a problem is essential, it is also necessary to know whether it is possible to modulate pathophysiological processes and thus potentially prevent fractures in diabetes, modulate possible hypoglycemia, which can lead to falls and fractures, and potentially reverse or prevent complications such as impaired eyesight, which can lead to an increased fracture risk.

We hope that you, too, will feel as inspired and enlightened as we did in editing this Research Topic.

## Author contributions

All authors have drafted and revised the editorial. All authors have approved the final version of the submitted editorial.

## Conflict of interest

The authors declare that the research was conducted in the absence of any commercial or financial relationships that could be construed as a potential conflict of interest.

## Publisher’s note

All claims expressed in this article are solely those of the authors and do not necessarily represent those of their affiliated organizations, or those of the publisher, the editors and the reviewers. Any product that may be evaluated in this article, or claim that may be made by its manufacturer, is not guaranteed or endorsed by the publisher.

